# Autophagy in Ovarian Cancer, an Opportunity or an Additional Threat?

**DOI:** 10.3390/ijms27104205

**Published:** 2026-05-09

**Authors:** Aleksandra Zoń, Ilona Anna Bednarek

**Affiliations:** Department of Biotechnology and Genetic Engineering, Faculty of Pharmaceutical Sciences in Sosnowiec, Medical University of Silesia, Jedności 8, 41-200 Sosnowiec, Poland

**Keywords:** autophagy, ovarian cancer, chemoresistance, cisplatin resistance

## Abstract

Despite extensive research, the treatment of ovarian cancer remains a significant challenge. One promising strategy involves the regulation of autophagy in cancer cells. However, this process is exceptionally complex and, depending on numerous factors, it can either suppress tumor progression, by maintaining genomic stability and limiting inflammatory responses, or exert a pro-tumor effect, enabling cancer cells to survive in unfavorable environmental conditions. Furthermore, the activation of this pathway can contribute to the development of resistance to drugs used in ovarian cancer therapy, such as cisplatin or paclitaxel. Current therapeutic approaches focus on both the induction and inhibition of autophagy, primarily by targeting the two most important pathways regulating this process: PI3K/Akt/mTOR and AMPK.

## 1. Introduction

For years, ovarian cancer (OC) has been a major oncological challenge among European women. It is estimated that in 2022, approximately 52,000 women in Europe were diagnosed with this cancer, and as many as 29,000 women succumbed to the disease [1]. Such a high mortality rate associated with OC is primarily attributed to its late-stage detection (caused mainly by the asymptomatic nature of early-stage disease and a lack of specific screening methods), as well as frequent recurrences and the development of drug resistance [2,3,4]. It is estimated that drug resistance occurs in up to 70% of patients treated with standard therapy methods, which typically involve surgical removal of the tumor followed by cyclic platinum-based chemotherapy. In drug-resistant cases, the recovery rate of OC is significantly reduced, as is indicated by the low 5-year survival rate, which stands at approximately 40% [2,5]. Consequently, there is currently an urgent need for novel therapeutic strategies that will provide more effective treatment of ovarian cancer. In this context, autophagy—a physiological process of “self-digestion”, responsible for the degradation of misfolded or damaged proteins and cellular organelles, has emerged as a potential target [6,7].

Autophagy is a process that occurs at a basal level in all eukaryotic cells, and plays a critical role in maintaining cell homeostasis. However this process can be upregulated by multiple stress factors such as nutrient deprivation, hypoxia, infection, increased levels of reactive oxygen species (ROS), growth factor deficiency, or exposure to cytotoxic agents, thereby promoting cell survival under unfavorable conditions [8,9].

In ovarian cancer, as in many other cancers, autophagy plays a dual role, and can exhibit both an inhibitory and a promoting effect on the development of cancer, depending primarily on its stage. Therefore, therapeutic strategies involving the modulation of autophagy are bidirectional, aiming for either its induction or inhibition [10].

This article discusses the multifaceted nature of autophagy as well as strategies and examples of its modulation in ovarian cancer therapy.

## 2. The Process of Autophagy and Its Regulation

The process of autophagy consists of six crucial stages: initiation, nucleation, elongation, autophagosome maturation, autophagosome and lysosome fusion, and cargo degradation [9,11].

During the initiation stage, the ULK1 complex, comprising three proteins: Unc-51 like autophagy activating kinase 1 (ULK1), Autophagy related 13 (ATG13), and FAK family kinase-interacting protein of 200 kDa (FIP200), is activated, and forms a structure near the endoplasmic reticulum (ER), that serves as a “scaffold” for the nascent autophagosome. Additionally, the active ULK1 complex phosphorylates and activates downstream proteins involved in the autophagy process. This step is regulated by two main signaling pathways: the PI3K/Akt/mTOR pathway (which inhibits initiation) and the AMPK pathway (which promotes initiation) [12,13,14].

The PI3K/Akt/mTOR pathway is a critical signaling network regulating key cellular processes, such as cell proliferation, growth, survival, and autophagy. It consists of three main components: phosphoinositide 3-kinase (PI3K), Akt serine/threonine kinase 1 (Akt), and mammalian target of rapamycin (mTOR) [15].

PI3K is a member of the lipid kinase family that acts as a serine/threonine kinase and a phosphatidylinositol kinase. PI3K activation (triggered in autophagy primarily by high cellular nutrient levels) leads to the phosphorylation of phosphatidylinositol-4,5-bisphosphate (PIP2), to form phosphatidylinositol-3,4,5-trisphosphate (PIP3), which acts as a secondary messenger [7,15]. PIP3 facilitates the phosphorylation of Akt at the Thr308 residue, thereby activating it, which consequently leads to the activation of mTOR kinase complex 1 (mTORC1) [7,15]. Active mTORC1 phosphorylates ULK1 at the Ser757 residue, preventing the activation of the ULK1 complex and, consequently, inhibiting autophagy initiation [12,14].

Conversely, under conditions of nutrient deprivation and a high AMP/ATP ratio, AMP-activated protein kinase (AMPK) is activated and promotes autophagy through two mechanisms: direct phosphorylation of ULK1 at Ser317 and Ser777, or activation of the Tuberous Sclerosis Complex 1/2, which results in the inactivation of mTORC1. Both pathways lead to the initiation of the autophagy process [14].

The subsequent step is nucleation, driven primarily by a complex of two proteins: Beclin-1 and phosphatidylinositol 3-kinase VPS34 (VPS34). This complex catalyzes the synthesis of phosphatidylinositol-3-phosphate, which recruits other proteins necessary for the later stages of autophagy [16]. The nucleation phase is regulated by the interaction of Beclin-1 with BCL family proteins. Its binding to BCL-2 and BCL-XL leads to the inhibition of autophagy, whereas its interaction with BH3-only proteins, such as BNIP3 and BAD, promotes autophagy [17,18].

The third and fourth stages, autophagosome elongation and maturation, are tightly regulated by two systems. The first is the ATG-12-ATG5-ATG16L1 protein complex, which participates in the formation of a scaffold, that facilitates autophagosomal membrane elongation [19,20]. The second system relies on Microtubule-associated protein 1A/1B-light chain 3 (LC3), which is cleaved by ATG4 to form LC3-I. LC3-I is then bound to phosphatidylethanolamine to generate LC3-II, which is later incorporated into the membrane of the expanding autophagosome, and participates in membrane elongation, cargo selection and autophagosome-lysosome fusion [21,22]. During cargo selection, ubiquitin-binding protein p62 (p62) acts as a receptor that binds ubiquitinated proteins and aggregates. By interacting with LC3-II, p62 docks the selected cargo within the autophagosome [23]. In the final stage, the autophagosome containing the selected cargo fuses with a lysosome to form an autolysosome. The engulfed cargo is then degraded by lysosomal hydrolases [24,25]. The fusion process is mediated by Ras-associated binding (RAB) proteins and soluble NSF attachment protein receptors (SNARE) proteins [26,27].

Finally, the breakdown products, such as amino acids, fatty acids, and other nutrients generated in the process of autophagic degradation, are released back into the cytoplasm for metabolic recycling [10].

Autophagy is modulated by numerous signaling pathways, including those regulated by genes frequently mutated in ovarian cancer.

A prominent example is the signaling pathway associated with the Tumor protein 53 (p53), which plays a crucial role in maintaining the genomic stability of cells. Following DNA damage, p53 inhibits the cell cycle at the G1/S or G2/M phase, allowing time for DNA repair. If the damage is irreversible, it directs the cell toward apoptosis, to prevent the propagation of mutations [28].

The p53 protein is encoded by the *TP53* gene. Mutations in this gene frequently result in the loss of tumor suppressor function or in the gain of oncogenic function. Approximately 47% of ovarian cancer patients harbor a *TP53* mutation [29].

Depending on its subcellular localization, environmental stressors, and mutational status, p53 can either activate or inhibit autophagy [30,31]. Under homeostatic conditions, wild-type p53 (wt-p53) is found primarily in the cytoplasm, where it exerts an inhibitory effect on autophagy [32]. However, upon exposure to stress, wt-p53 translocates to the nucleus, where it accumulates and induces autophagy. Wild-type p53 can induce autophagy through several different mechanisms, including the inhibition of mTORC1 via the upregulation of PI3K/Akt pathway inhibitors, such as Phosphatase and tensin homolog (PTEN). Furthermore, wt-p53 can increase the expression of AMPK activators, such as Sestrin 1 and 2, leading to AMPK-mediated initiation of autophagy [30,33].

Moreover, wt-p53 has the ability to upregulate the expression of autophagy-related genes, including *DRAM1* (DNA damage-regulated autophagy modulator 1), which is crucial for lysosomal acidification and enzyme activation, *ULK1,* or *ATG* family genes [30,34,35,36].

Conversely, mutant p53 (particularly the “gain-of-function” mutants), can suppress autophagy by attenuating AMPK activity or downregulating the expression of key autophagic genes such as *BECN1*, *DRAM1*, or *ATG12* [37,38]. This p53 mutant-mediated inhibition likely provides a survival advantage to cancer cells by allowing them to evade autophagic cell death [30].

Another example of an autophagy-modulating network is the RAS/MAPK pathway, which involves the Kirsten rat sarcoma viral oncogene homolog protein (K-RAS). As a small GTPase, K-RAS acts as an “on/off switch” that transmits extracellular signals from receptors into the cell. K-RAS can exist in two forms: an inactive form when bound to guanosine diphosphate (GDP), and in an active, signal-transmitting form, when bound to guanosine triphosphate (GTP). The activation and deactivation processes are tightly regulated by Guanine nucleotide exchange factors (GEFs), which promote activation, and GTPase-activating proteins (GAPs), which facilitate deactivation. Active K-RAS stimulates major downstream pathways, including RAF/MEK/ERK and PI3K/Akt/mTOR, thereby governing proliferation, growth, differentiation, migration, apoptosis, and autophagy [39,40].

Statistics show that mutations in the *KRAS* gene, occur in approximately 14% of ovarian cancer cases [29]. These mutations typically result in the formation of a K-RAS protein that has a limited ability to bind to GTPase-activating proteins, leading to the inhibition of GTP hydrolysis and causing K-RAS to remain in its active form [41]. This persistent activation drives uncontrolled proliferation, inhibits apoptosis, and enhances the invasive potential of cancer cells [41].

In normal cells, K-RAS inhibits autophagy by activating the PI3K/Akt pathway, leading to mTORC1 activation and subsequent ULK1 phosphorylation [42]. However, in cancer cells with a *KRAS* gene mutation, the dysfunctional protein induces autophagy by activating AMPK and increasing the expression of autophagy-related proteins such as ATG5. In this context, higher levels of autophagy in cancer cells serve as an adaptive mechanism, enabling cancer cells to endure metabolic stress and nutrient deprivation, thus promoting tumor survival and progression [42,43].

Finally, another vital regulator of autophagy is the PTEN pathway. PTEN is a phosphatase that acts as a primary negative regulator of the PI3K/Akt/mTOR cascade by dephosphorylating PIP3 [44,45]. Through this inhibition, wild-type PTEN functions as a tumor suppressor, preventing the growth, proliferation, and survival of cancer cells [46]. Furthermore, when located in the cell nucleus, PTEN interacts with p53 to regulate the cell cycle and binds to centromere protein C to prevent premature centromere separation, thereby maintaining chromosomal stability [47,48]. Mutations in the *PTEN* gene, which occur in 5% of ovarian cancer patients, result in the loss of its suppressor function, leading to the development of cancer [29].

As mentioned earlier, as wild-type PTEN inhibits the PI3K/Akt/mTOR pathway, it indirectly promotes autophagy [49]. The loss of PTEN function leads to the inhibition of autophagy, caused by the high activity of the PI3K/Akt/mTOR pathway, increasing the survival of cancer cells [50].

## 3. A Dual Role of Autophagy in Ovarian Cancer

### 3.1. Early Stages of Ovarian Cancer—Inhibition of Tumor Development

**Figure 1:**
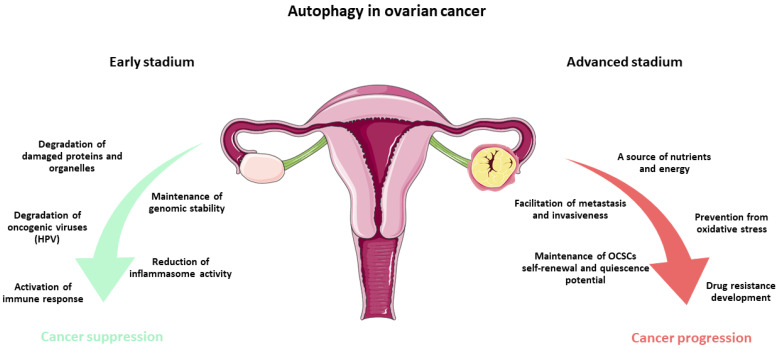
Mechanisms of autophagy in the inhibition and progression of ovarian cancer: A summary based on the stage of disease development. The elements used in the graphic have been adapted from Servier Medical Art (https://smart.servier.com/), licensed under CC BY 4.0 (https://creativecommons.org/licenses/by/4.0/ (accessed on 27 March 2026)).

During the early stages of ovarian cancer development, autophagy can inhibit tumorigenesis through several distinct mechanisms. A summary of the anti-cancer effects of autophagy is presented in Figure 1.

The most important mechanism of tumorigenesis inhibition mediated by autophagy is the maintenance of cellular homeostasis through the degradation of damaged or misfolded proteins and defective organelles, primarily mitochondria [51]. Removing the latter is particularly important because damaged or malfunctioning mitochondria produce excessive amounts of reactive oxygen species, which can cause oxidative damage to the cell’s genetic material. Therefore, the autophagic degradation of these structures facilitates, to some extent, the maintenance of genomic stability, preventing neoplastic transformation [51]. Autophagy is also involved in the removal of protein aggregates, which might disrupt the proper function of signal transduction pathways and promote oncogenesis [52,53]. Studies have demonstrated that cells with low autophagic activity accumulate DNA damage, including double-stranded breaks and centrosome abnormalities, leading to chromosomal instability and subsequent neoplastic transformation [54].

Another important mechanism of autophagy-mediated tumorigenesis inhibition is the reduction of inflammasome activity. Inflammasomes are protein complexes that upon contact with an antigen, induce the production of proinflammatory cytokines, including interleukins 1α, 1β and 18, thereby activating the inflammatory response. Dysregulation of this process may lead to the development of chronic inflammation, which is considered an important carcinogenesis factor [55]. By eliminating cellular components damaged during infection, autophagy contributes to the inhibition of inflammasome activity, primarily the NLR family pyrin domain containing 3 (NLRP3) inflammasome, thus restraining inflammatory responses and, consequently, reducing the probability of cancer initiation [55,56].

Autophagy can also prevent carcinogenesis indirectly, by degrading oncogenic viruses, the most important in the context of OC is the human papillomavirus (HPV). The oncogenic activity of HPV is driven by two proteins: E6 and E7, which promote the neoplastic transformation of infected cells, and tumor growth. By targeting p53 for degradation, as well as by inhibiting the expression of cyclin-dependent kinase inhibitors such as Cyclin-dependent kinase inhibitor 1 (p21) and Cyclin-dependent kinase inhibitor p27 (p27), the E6 protein disrupts the proper function of cell cycle checkpoints, leading to uncontrolled cell proliferation. Additionally, this protein can activate human telomerase reverse transcriptase (hTERT) leading to cellular immortalization. In parallel, the E7 protein has the ability to bind to retinoblastoma protein (pRB), causing the release of the E2F transcription factor, which allows cells to enter the S phase of the cell cycle, contributing to their uncontrolled proliferation [57,58,59]. Currently, the link between HPV infection and the development of ovarian cancer has not been conclusively confirmed, however it is estimated that approximately 20% of OC patients are infected with HPV [60].

Moreover, during the early stages of cancer development, autophagy can also facilitate immune cell activation [61]. During the autophagic death of cancer cells, the release of damage-associated molecular patterns (DAMPs), primarily High Mobility Group Box 1 (HMGB1), can occur. The HMGB1 molecule attracts T lymphocytes and dendritic cells to the vicinity of the tumor, thereby activating anti-tumor immunity [62,63].

Another way by which autophagy exerts a suppressive effect on OC development is its ability to inhibit the process of cancer cell metastasis at its early stages. One of the mechanisms by which autophagy suppresses the epithelial–mesenchymal transition (EMT) in cancer cells is likely through a pathway associated with ROS and heme oxygenase-1 (HO-1) [61,64]. The link between the activation of epithelial–mesenchymal transition and autophagy inhibition has also been demonstrated through the downregulation of the ubiquitin-conjugating enzyme E2T (UBE2T) expression. UBE2T is a key component of the ubiquitin-proteasome system involved in DNA damage repair, cell cycle regulation, and cell apoptosis [65]. The knockout of the *UBE2T* gene leads to decreased activity of the Akt/mTOR signaling pathway, subsequent activation of autophagy, and inhibition of EMT, consequently limiting the proliferation and invasiveness of ovarian cancer cells [65].

The suppressive role of autophagy in the early-stage of OC development is supported by research indicating the presence of a monoallelic deletion of the *BECN1* gene in approximately 50% of patients with this cancer. As mentioned earlier, Beclin-1 is one of the most important regulatory proteins in the autophagy process, controlling its nucleation stage [16]. The significantly higher incidence of ovarian, breast, and prostate cancers in heterozygotes with a monoallelic deletion of *BECN1*, compared to homozygotes with two copies of that gene, indicates that it is a haploinsufficient tumor suppressor.

In vivo studies have shown that mice with a monoallelic deletion of *BECN1* are more prone to spontaneous neoplastic transformation, increased cell proliferation, and decreased autophagy levels compared to wild-type mice [66].

These findings correlate with clinical statistics showing that reduced levels of Beclin-1 in OC patients correlates with shorter progression-free survival and overall survival, acting as a poor prognostic factor in OC [67,68].

### 3.2. Advanced Stages of Ovarian Cancer—Promotion of Tumor Development

In the advanced stages of ovarian cancer, autophagy plays a diametrically opposite role, contributing to and facilitating further disease progression. The summary of the pro-cancer mechanisms of autophagy is presented in Figure 1.

Primarily, an increased level of autophagy allows cancer cells to adapt to unfavorable environmental conditions, such as nutrient deprivation, low pH, or hypoxia [69].

The most important energy source for cancer cells is glucose. Its deficiency in the tumor microenvironment causes hexokinase II (the enzyme catalyzing the first step of glycolysis—the phosphorylation of glucose to glucose-6-phosphate) to bind to mTORC1. This interaction reduces the complex’s activity and, consequently, induces autophagy in OC cells, thus providing an alternative source of nutrients [70,71]. Increased levels of autophagy may also occur in response to amino acid and lipid deficiency, providing cancer cells with metabolic plasticity. A reduction in the amino acid levels leads to the increased expression of GTP-binding protein Di-Ras3 (DIRAS3), which induces autophagy by inhibiting the PI3K/Akt/mTOR signaling pathway [72]. Under lipid deficiency conditions, ovarian cancer cells activate lipophagy, a selective autophagy process involving the degradation of intracellularly stored lipid droplets. This results in the release of free fatty acids, which are a rich source of energy necessary for further tumor development [73,74].

Uncontrolled proliferation and the rapid expansion of tumor tissue necessitate enhanced angiogenesis. Accelerated angiogenesis often results in the formation of irregular, chaotic, and leaky blood vessels, ultimately leading to impaired blood supply resulting in hypoxia [75]. In hypoxic conditions, cancer cells experience an imbalance between the production of ROS and antioxidants—a condition known as oxidative stress. Excessive ROS formation can cause irreversible oxidative damage to cellular components, potentially leading to cell death [76,77]. One of the mechanisms developed by cancer cells to evade the accumulation of damaged cellular structures is increased autophagy. Similar to its role in normal cells, an increase in autophagy levels in cancer cells leads to the removal of dysfunctional organelles, such as mitochondria, peroxisomes, and lysosomes, as well as protein aggregates. This prevents the accumulation of ROS and further oxidative damage to cancer cells [77,78].

Increased autophagy can also promote OC progression by facilitating its metastasis to the peritoneal cavity. It has been shown that OC cells that are capable of metastasizing and surviving in the peritoneal microenvironment exhibit elevated levels of autophagy, which plays a protective role under stressful conditions [79].

The precise mechanism underlying the effect of autophagy on the metastatic potential of OC cells is not yet fully understood. However, based on the studies of other types of cancer, it can be assumed that autophagy influences the ability of cancer cells to avoid anoikis (a type of cell death caused by the detachment from the matrix), and to undergo epithelial–mesenchymal transition [80,81]. This may be supported by the involvement of autophagy-related proteins in these processes. For example, during anoikis, a significant increase in the expression of autophagy-related proteins ATG5 and LC3B can be observed. Furthermore, the autophagic receptor p62, demonstrates the ability to bind, and stabilize the TWIST protein, which is a regulator of the EMT process. The stabilization of TWIST inhibits its proteasomal degradation, leading to the intensification of EMT, and consequently, to increased invasiveness of rat bladder cancer cells [81,82].

Additionally, autophagy can enhance the invasiveness of OC cells by promoting the formation of invadopodia—specialized cell structures that exhibit proteolytic activity against extracellular matrix components, enabling cell migration into adjacent tissues. In ovarian cancer cells, under stress conditions, p62 activates the Extracellular Signal-Regulated Kinase 1 and 2 (ERK1/2), which phosphorylates cortactin, a protein essential for invadopodia formation, thus contributing to increased invasiveness [83,84].

The process of autophagy is also utilized by ovarian cancer stem cells (OCSCs) to maintain their self-renewal and quiescence potential. OCSCs are a small population of cancer cells capable of self-renewal and differentiation that play a significant role in metastasis and cancer cell proliferation, often contributing to disease relapse and treatment resistance [85]. It has been shown that autophagy allows OCSCs to remain in a quiescent state, and then to reenter the cell cycle in response to different stimuli. Furthermore, by maintaining low levels of ROS in OCSCs, autophagy enables their self-renewal by preserving high expression levels of the Neurogenic locus notch homolog protein 1 (NOTCH1) gene, which regulates cell differentiation, proliferation, and survival [86,87].

## 4. Autophagy in the Development of Drug Resistance in Ovarian Cancer Treatment

For many years, the standard treatment for ovarian cancer has been based on chemotherapy involving platinum compounds (such as cisplatin and carboplatin) in combination with taxanes (primarily paclitaxel and docetaxel), administered in various doses and regimens. Additionally, some patients are treated with bevacizumab, a monoclonal antibody that binds vascular endothelial growth factor (VEGF) and inhibits tumor angiogenesis, while patients with Breast cancer type 1/2 susceptibility (BRCA1/2) gene mutations undergo treatment with inhibitors of poly-ADP-ribose polymerase (PARP), an enzyme involved in the repair of single-stranded DNA breaks [88,89].

Approximately 70% of patients undergoing OC therapy develop resistance to the administered drugs, which results in recurrence of the disease with a poor prognosis. Cisplatin resistance is the most common cause of treatment failure, although some patients may also develop resistance to other drugs [90,91,92].

There are two types of drug resistance: intrinsic and acquired. Intrinsic drug resistance occurs despite no prior exposure to the drug and can be caused, for example, by impairments in transmembrane drug transport mechanisms (e.g., the activity of ATP-binding cassette (ABC) transporters), the presence of drug-degrading enzymes (e.g., cytochrome P450, glutathione S-transferases), or mutations at the drug’s target site (e.g., the T790M mutation in the epidermal growth factor receptor (EGFR) gene in patients with non-small cell lung cancer) [4,93]. Acquired resistance arises from mutations and changes that occur in cancer cells following drug exposure. The most significant mechanisms causing acquired resistance include changes in the activity of cellular transporters regulating drug influx and efflux, increased activation of DNA damage repair mechanisms, inhibition of apoptosis, and changes in autophagy [4,93].

The process of autophagy is involved in the development of resistance to the most important drugs used in ovarian cancer treatment—cisplatin, paclitaxel, and PARP inhibitors [94,95,96].

Currently, the most important drug in OC therapy is cisplatin. Its main mechanism of action involves binding to the nitrogenous bases in DNA, leading to the formation of DNA-drug adducts that damage the cell’s genetic material and, consequently, drive the cell towards apoptosis. Additionally, by increasing the production of free oxygen radicals in cancer cells, cisplatin induces oxidative stress in these cells [4,97].

One way in which autophagy can contribute to cisplatin resistance is through the removal of misfolded or damaged proteins from cancer cells. These proteins can be formed through direct binding to cisplatin (particularly proteins rich in sulfur-containing amino acids and those containing histidine), as well as through translation errors caused by the formation of cisplatin-DNA adducts. Protein damage can also results from the action of free oxygen radicals generated by cisplatin-induced oxidative stress [98]. It has been demonstrated that p62 has the ability to bind these abnormal proteins and transport them to the autophagosome for degradation, allowing cancer cells to avoid the accumulation of protein aggregates that would otherwise result in endoplasmic reticulum stress. Removal of damaged proteins by autophagy, therefore, protects cells from cisplatin-induced, ER stress-related apoptosis [99].

Autophagy can also mediate the repair of DNA damage induced by cisplatin. Exposure of OC cells to cisplatin causes the activation of AMPK, and consequently, the phosphorylation of Beclin-1, which, by dissociating from BCL-2, induces subsequent stages of the autophagy process. Increased levels of autophagy in cancer cells result in a decrease in the amount of free p62, preventing it from binding to the ubiquitin ligase Ring finger protein 168 (RNF168). Free RNF168 ligase is then transported to the cell nucleus, where it participates in the ubiquitination of histone H2A. Ubiquitination of this histone enables the recruitment of proteins involved in the repair of double-stranded DNA breaks, including DNA repair protein RAD51 homolog 1 (RAD51), Breast cancer type 1 susceptibility protein (BRCA1), and Receptor-associated protein 80 (RAP80). This, in turn, leads to the activation of DNA damage repair processes, enabling OC cells to avoid cisplatin-induced apoptosis [100].

In addition to binding to nuclear DNA, cisplatin can also form adducts with mitochondrial DNA, leading to disturbances in the expression of mitochondrial genes, which results in improper functioning of mitochondria, increased production of ROS in cancer cells, and cell death by oxidative stress-induced apoptosis [101].

Compared to cisplatin-sensitive cells, cisplatin-resistant OC cells exhibit higher levels of mitophagy (selective autophagy of mitochondria). This suggests that an increased rate of mitochondrial turnover through autophagy allows cancer cells to effectively remove cisplatin-damaged mitochondria, thereby preventing the excessive generation of ROS and oxidative stress [102].

Paclitaxel was introduced into OC therapy in the 1990s. Its mechanism of action is based on the inhibition of cancer cell division by binding to microtubules—specialized structures forming the mitotic spindle, which is essential for chromosome segregation during mitosis. By binding to the β-subunit of tubulin, paclitaxel stabilizes microtubules and prevents their depolymerization, thus arresting cells in the G2/M cell cycle phase and leading to their apoptosis [93,103].

One of the proposed mechanisms for paclitaxel resistance is the upregulation of autophagy in cells exposed to this drug. In paclitaxel-treated SKOV-3 ovarian cancer cells, increased expression of Thioredoxin domain-containing protein 17 (TXNDC17) is observed. TXNDC17 is a disulfide reductase involved in the Tumor necrosis factor signaling pathway, which activates autophagy by interacting with Beclin-1 via the Nuclear Factor Kappa B Subunit 1 (NFKB1) signaling pathway. Autophagy activation leads to a decrease in the cytotoxic effect of paclitaxel and increases the survival of SKOV-3 ovarian cancer cells [93,95].

The newest class of drugs in OC treatment comprises PARP inhibitors, which can be used in the therapy of patients with *BRCA1/2* mutations or other homologous recombination deficiencies. The mechanism of anticancer action of PARP inhibitors is based on the inhibition of poly(ADP-ribose) polymerase, an enzyme involved in the repair of single-stranded DNA breaks, which frequently occur in rapidly proliferating cells. Failure to repair single-stranded DNA breaks, leads to the formation of double-stranded DNA breaks, which can only be repaired through homologous recombination. In cells deficient in homologous recombination, this repair is impossible, which ultimately leads to cell death [104,105].

In PARP-resistant ovarian cancer cells, the formation of double-stranded DNA breaks triggers the activation of PTEN phosphatase. PTEN inhibits the mTOR signaling pathway, consequently leading to the activation of the autophagy process, which acts as an adaptive mechanism of resistance to this group of drugs [96].

## 5. Autophagy as a Therapeutic Target

Due to the complex nature of autophagy, the regulation of this process in ovarian cancer therapy is highly difficult, therefore current attempts are not limited to a single strategy, but include both the activation and inhibition of this process [106] Examples of autophagy regulation mechanisms in ovarian cancer therapy are summarized in Table 1.

### 5.1. Activation of the Autophagy Process

The most important mechanism of autophagy activation in ovarian cancer treatment is the inhibition of the PI3K/Akt/mTOR signaling pathway.

This mechanism is exhibited by the natural compound—cardamonin, a chalcone found in *Elettaria cardamomum* L. By downregulating hexokinase II and inhibiting glycolysis in SKOV-3 ovarian cancer cells, this substance inhibits mTOR kinase activity, which consequently leads to the induction of autophagy in OC cells, contributing to cardamonin’s potent antiproliferative effect on SKOV-3 cells [107].

A similar mechanism of action is shown by another plant-derived substance—harmine, a β-carboline alkaloid found in *Peganum harmala* L. This substance strongly inhibits the proliferative and metastatic potential of SKOV-3 ovarian cancer cells by promoting autophagy through the inhibition of the PI3K/Akt/mTOR pathway [108].

The strategy of PI3K/Akt/mTOR pathway inhibition is also utilized in combination therapy with EGFR inhibitor—erlotinib, and PARP inhibitor—olaparib, as confirmed by the results of in vitro studies on A2780 ovarian cancer cells and in vivo studies on xenografts derived from these cells in BALB/c nude mice. Therapy with both of these drugs inhibits the activity of Akt kinase, which results in the activation of autophagy and, consequently leads to the inhibition of tumor xenograft growth [109].

Activation of autophagy is also attempted by inducing the expression of proteins involved in this process. An example of this strategy is the use of ginsenoside 20(S)-Rg3, a triterpene saponin that is an active ingredient in *Panax ginseng*. By increasing the expression of ATG5, ATG7, and LC3 proteins, ginsenoside 20(S)-Rg3 induces autophagy in SKOV-3 ovarian cancer cells, leading to the inhibition of their migration and invasion in vitro [110].

Intensification of autophagy through the upregulation of proteins involved in this process, namely LC3II, ATG3, ATG4B, and Beclin-1, is a mechanism also demonstrated by farletuzumab (MORAB-003), a humanized monoclonal antibody targeting folate receptor alpha (FRα). Blocking FRα by MORAB-003 in OC cells expressing high levels of this receptor leads to cell death due to prolonged autophagy, contributing to the inhibition of tumor growth [111].

Another potential mechanism for autophagy induction that might be used in OC therapy, is the increase of ROS production in cancer cells. An example of this strategy is the use of triptolide, a diterpene compound isolated from the Chinese plant *Tripterygium wilfordii*. Triptolide has the ability to inhibit the growth of cisplatin-resistant SKOV-3 ovarian cancer cells by enhancing autophagy. Exposure of OC cells to this compound results in increased production of free oxygen radicals. High levels of ROS inhibit the JAK2/STAT3 signaling pathway, which is responsible for regulating the transcription of many genes, including those involved in autophagy. Inhibition of the JAK2/STAT3 pathway blocks the expression of Myeloid Cell Leukemia-1 (MCL-1), a member of the BCL protein family, that can bind to Beclin-1, and thus block autophagy. A lower level of MCL-1 inhibits its interaction with Beclin-1, leading to the release of Beclin-1 and, consequently, to the induction of autophagy in treated cells [112].

Activation of autophagy by increasing the production of ROS in cancer cells is also the mechanism exhibited by apatinib, a tyrosine kinase inhibitor capable of inhibiting the growth and migration of ovarian cancer cells. Apatinib suppresses the activity of the NRF2/HO-1 system, which is responsible for protecting cells from oxidative stress. Nuclear factor erythroid 2-related factor 2 (NRF2) is one of the most important regulators of the cellular response to oxidative stress. Under homeostatic conditions, NRF2 binds to kelch-like ECH-associated protein 1 (KEAP1) which inhibits its activity. As a result of increased levels of ROS in cells, NRF2 dissociates from KEAP1 and is translocated into the nucleus, where it activates the expression of genes encoding antioxidant and cytoprotective proteins. One of these genes is *HMOX1*, which encodes heme oxygenase-1—an enzyme that catalyzes the first step in heme degradation, producing carbon monoxide, iron, and biliverdin, which is then broken down into bilirubin. The products of this reaction have potent antioxidant and anti-inflammatory properties [113,114]. Repressing the activity of the NRF2/HO-1 system, results in a reduction in the levels of intracellular antioxidants in cancer cells, leading to an increase in ROS levels, which in turn results in the activation of autophagy and apoptosis processes induced by oxidative stress [114].

Induction of autophagy can also be achieved by activating the pathways responsible for the initiation of this process. This mechanism is used by neferine, an alkaloid found in the green seed embryos of *Nelumbo nucifera* Gaertn., as well as by eclalbasaponin, a saponin isolated from *Eclipta prostrata*. These substances, activate the p38 MAPK/JNK signaling pathway, leading to the inhibition of mTORC1 and, consequently, to the intensification of autophagy processes and the inhibition of A2780 OC cell growth [115,116].

Autophagy can also be regulated indirectly by modulating the expression of its regulatory proteins. An example of this mechanism is the use of dasatinib, an inhibitor of SRC/ABL tyrosine kinases. Dasatinib inhibits the proliferation and colony formation of SKOV-3 and HAY ovarian cancer cells by reducing the expression of BCL-2, which acts as a negative regulator of autophagy. A reduction in the amount of this protein leads to the inhibition of its interaction with Beclin-1, thus contributing to the induction of autophagy [117].

### 5.2. Inhibition of the Autophagy Process

The most important strategy of autophagy suppression is based on the activation of the PI3K/Akt/mTOR signaling pathway, which is responsible for inhibiting this process. This mechanism is demonstrated by two compounds used in the studies on ovarian cancer treatment—icariin and nobiletin. Icariin is a flavonoid found in perennial plants of the Berberidaceae family. Its ability to activate the Akt/mTOR signaling pathway inhibits autophagy in CKVCR ovarian cancer cells, thereby increasing their sensitivity to the cytotoxic effects of cisplatin [118]. In turn, nobiletin, a flavone glycoside abundant in citrus fruit peels, inhibits autophagy in paclitaxel-resistant SKOV-3 cells, through the activation of Akt kinase, leading to the inhibition of their growth and proliferation in vitro [119].

Another strategy for inhibiting autophagy in ovarian cancer cells, is the downregulation of key autophagy-related proteins expression. An example of such a strategy is the silencing of the Activating Molecule in Beclin-1-Regulated Autophagy 1 (AMBRA1) gene by RNA interference using short hairpin RNA (shRNA). AMBRA1 is an autophagy regulatory protein that, by interacting with Beclin-1, participates in the regulation of autophagosome formation. Inhibition of *AMBRA1* expression in OVCAR-3 cells, leads to a reduction in autophagy activation and, consequently, contributes to increased cisplatin-sensitivity [120].

An interesting mechanism of autophagy inhibition is demonstrated by NEO212, an alkylating agent and temozolomide analog that inhibits the proliferation of ovarian cancer cell lines SKOV-3, OVCAR-3, and A2780 in vitro. NEO212 has the ability to enhance the phosphorylation of the ERK and Akt kinase, which regulate the transport of transcription factor EB (TFEB) into the cell nucleus. TFEB controls the transcription of genes involved in the formation and maturation of lysosomes, including genes encoding lysosomal hydrolases, proteins that build lysosomal membranes, and enzymes responsible for lysosomal acidification [121]. The phosphorylation of TFEB by ERK and Akt kinases inhibits its transport into the cell nucleus, leading to impaired lysosome production in cancer cells. A reduction in the number of lysosomes leads to the accumulation of autophagosomes within the cell and, consequently, induces apoptosis [121].

A similar mechanism of action is exhibited by elaiophyllin, a macrodiolide antibiotic produced by *Streptomyces melanosporus*. This substance inhibits autophagy by decreasing the activity of lysosomal cathepsins B and D, and by damaging lysosomal membranes, contributing to the induction of SKOV-3 cell death via apoptosis [122].

**Table 1:** Summary of compounds involved in the regulation of autophagy in ovarian cancer treatment.

Compound	Effect on Autophagy	Mechanism of Action	Study Status	Reference
Cardamonin	Activation	Downregulation of hexokinase II and inhibition of glycolysis	Preclinical	[107]
Harmine	Activation	Inhibition of PI3K/Akt/mTOR pathway		[108]
Erlotinib + Olaparib	Activation	Inhibition of Akt kinase		[109]
Ginsenoside 20(S)-Rg3	Activation	Upregulation of ATG5, ATG7, LC3 expression		[110]
Farletuzumab	Activation	Upregulation of LC3II, ATG3, ATG4B, Beclin-1, expression		[111]
Triptolide	Activation	Elevation of ROS production		[112]
Apatinib	Activation	Repression of the NRF2/HO-1 system		[114]
Neferine	Activation	Activation of p38 MAPK/JNK signaling pathway		[115]
Eclalbasaponin	Activation	Activation of p38 MAPK/JNK signaling pathway		[116]
Dasatinib	Activation	Reduction in the expression of BCL-2		[117]
Icariin	Inhibition	Activation of Akt/mTOR signaling pathway		[118]
Nobiletin	Inhibition	Activation of Akt kinase		[119]
NEO212	Inhibition	Impaired lysosome production		[121]
Elaiophyllin	Inhibition	Inhibition of lysosomal cathepsins B and D activity		[122]
Chloroquine	Inhibition	Inhibition of the autophagosome-lysosome fusion	Preclinical	[123,124]
Hydroxychloroquine	Inhibition	Inhibition of the autophagosome-lysosome fusion	Phases I and II	[125,126]
MRT68921	Inhibition	Inhibition of ULK1		[127]
DCC-3116 (Inlexisertib)	Inhibition	Inhibition of ULK1/2	Phase I/II	[128]

## 6. Current Status and Future Perspectives

Currently, attempts to utilize autophagy modulation in the treatment of ovarian cancer patients primarily involve inhibiting this process and focus on the use of chloroquine and its derivative, hydroxychloroquine. The mechanisms of action of both substances consist of blocking the fusion of the autophagosome with the lysosome, thereby inhibiting autophagy [129].

Intensive efforts to apply these substances in ovarian cancer therapy are reflected in ongoing clinical trials. A notable example is the Phase I/II HYDRA-1 trial (NCT03081702), which aimed to evaluate the safety, tolerability, and preliminary efficacy of itraconazole and hydroxychloroquine therapy in patients with epithelial ovarian cancer. The results of the study indicated that the use of these drugs at the tested doses is safe, although the study did not demonstrate clinically significant antitumor activity [125]. An attempt at autophagy inhibition was also undertaken in the Phase II trial CTRI/2020/06/025790, which investigated the potential use of hydroxychloroquine in combination with standard chemotherapy regimens (carboplatin with paclitaxel/gemcitabine) for patients with platinum-sensitive recurrent ovarian cancer. Unfortunately, in this case as well, the results did not indicate significant benefits from the use of hydroxychloroquine [126].

Currently, a Phase II clinical trial is underway evaluating the potential use of CPI-613 (devimistat) in combination with hydroxychloroquine and either 5-fluorouracil or gemcitabine in patients with advanced or chemoresistant solid tumors, including ovarian cancer (NCT05733000) [130]. Additionally, another Phase II study—Autophagy Maintenance (AUTOMAIN, NCT06971744) is being conducted to determine whether the use of hydroxychloroquine and nelfinavir mesylate in combination with bevacizumab will serve as a beneficial maintenance treatment for ovarian cancer patients [131].

A significant drawback of chloroquine and hydroxychloroquine is the fact that they act at a late stage of the autophagy (autophagosome-lysosome fusion), which is why they are considered non-specific inhibitors. Therefore, a crucial direction for autophagy modulation is its targeted regulation at the initiation stage. Currently, ULK1 kinase inhibitors are garnering the most attention, as they enable the inhibition of autophagy at its initiation stage. An example of such an inhibitor is MRT68921, which in in vitro studies demonstrated the ability to inhibit autophagy, leading to a significant reduction in the viability of ovarian cancer cells [127]. Another ULK1 kinase inhibitor, DCC-3116 (inlexisertib), is also currently in Phase I/II clinical trials for patients with advanced or metastatic solid tumors [131].

**Figure 2:**
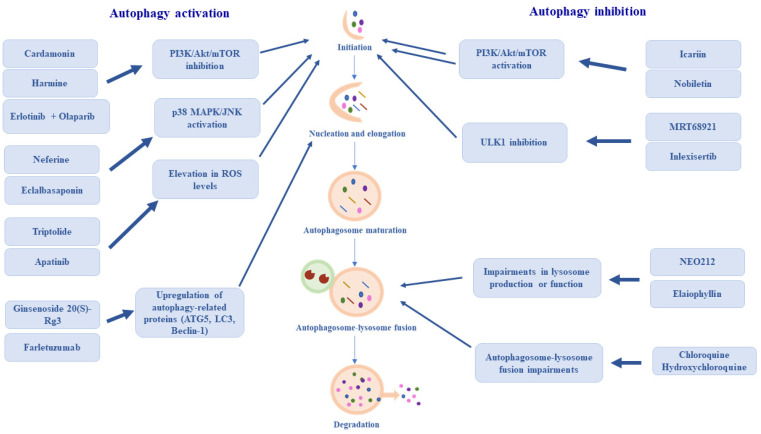
An overview of current investigational compounds and their molecular mechanisms in the treatment of ovarian cancer.

The currently investigated compounds that modulate the autophagy process, along with their mechanisms of action, are summarized in Figure 2.

A slightly different approach to utilizing autophagy in the treatment of ovarian cancer involves nanotherapy using, for example, peanut-shaped gold nanoparticles. It has been demonstrated that treating SKOV-3 ovarian cancer cells with these nanoparticles leads to increased ROS production and a simultaneous reduction in intracellular glutathione levels, thereby inducing oxidative stress in these cells. Consequently, autophagy and apoptosis are triggered in SKOV-3 cells, leading to a decrease in their viability [132].

Since autophagy significantly contributes to the development of ovarian cancer cell resistance, not only to typical drugs such as cisplatin but also to modern therapies, including PARP inhibitors, research into combination therapies of autophagy inhibitors with these drugs certainly represents an important future direction. An example of such therapy is the use of the long noncoding RNA molecule LOC730101, which, by binding to the key autophagy protein Beclin-1, inhibits its dissociation from Bcl-2. This, in turn, prevents the formation of a complex with the VPS34 protein and the subsequent activation of autophagy. As described in Chapter 4, autophagy can mediate the repair of single-strand DNA breaks, therefore, its inhibition by LOC730101 increases the sensitivity of cancer cells to PARP inhibitors, such as niraparib [100].

A particularly important research direction involves not only autophagy itself but also the identification of biomarkers for its effective monitoring in ovarian cancer patients. Currently, autophagy levels are monitored by assessing the levels of autophagy-related proteins, primarily LC3 and p62, through immunoblotting [133]. The use of LC3 as a autophagy biomarker is based on its conversion into LC3-II during autophagosome maturation. Consequently, an increase in LC3-II levels can be interpreted as an increase in the number of autophagosomes, and as enhanced autophagy. However, a significant limitation of this biomarker is that LC3-II levels also rise when autophagy is blocked and autophagosome degradation is inhibited [134,135].

Similarly, p62 is utilized in autophagy monitoring, as its decrease correlates with its increased degradation during the autophagic process. Nevertheless, an increase in p62 is not specific to autophagy, as it can also be induced by various stress factors [134,135].

The most significant drawback of these biomarkers is that they provide a static measurement of proteins level, which frequently leads to inaccurate interpretations. To precisely determine autophagy activity, it is necessary to assess the dynamic autophagic flux, however, this would require frequent, invasive biopsies, which are impractical for patients [134,135]. Consequently, it is essential to identify novel markers and develop non-invasive methods of obtaining samples, such as the analysis of ascites fluid [136].

## 7. Summary

Despite many years of intensive research, effective therapy for ovarian cancer remains a challenge. In 1999, after Beth Levine and her team identified *BECN1* as a tumor suppressor gene, and thus established the link between autophagy and cancers, autophagy emerged as another promising treatment strategy for many cancers, including ovarian cancer. Unfortunately, over the years and with subsequent research, it turned out that autophagy is a process of a dual nature, which can be a major obstacle to utilizing it in systemic treatment of cancers, leaving the question from our paper title open for discussion.

Undoubtedly, one of the significant obstacles to utilizing autophagy in cancers therapy, including ovarian cancer, is tumor heterogeneity, characterized by cellular diversity within the tumor. In poorly vascularized regions, characterized by hypoxia and nutrient deficiency, cells exhibit high levels of autophagy, which serves them as a survival mechanism. Conversely, in well-vascularized regions, cancer cells do not demonstrate such a strong dependence on the autophagy process. Given the presence of these highly diverse cell populations, modulating autophagy will be effective only against a subset of them, while its impact on the remainder will be minimal [137].

Furthermore, it is important to note that the systemic administration of autophagy-based therapies is not without impact on normal cells. This necessitates further research into drug carriers that would enable the precise targeting and release of these compounds within the tumor site [138].

While the in vitro evidences regarding the modulation of autophagy in OC treatment are promising, many challenges remain in translating these findings into clinical practice. Currently, the majority of the data are derived from in vitro models, particularly SKOV-3 and A2780 cell lines. While these models are essential during preclinical studies, they may not fully encapsulate the complexity of ovarian tumors. This is reflected in clinical trials using hydroxychloroquine, the results of which do not indicate significant benefits of using this drug in patients. Therefore, further clinical trials are necessary to determine whether it is possible to achieve satisfactory results also in vivo.

Taking everything into consideration, the “double-edged sword” nature of autophagy remains a significant therapeutic problem. As demonstrated earlier in the article, autophagy can either promote cell survival or induce cell death, depending mostly on stage of tumor progression, but also other variables. This dependency suggests that a single approach to autophagy regulation is likely to fail. Instead, successful therapy will require precise determination whether a patient would benefit more from autophagy inhibition or activation.

## Data Availability

No new data were created or analyzed in this study. Data sharing is not applicable to this article.

## Abbreviations

ABC	ATP-binding cassette transporter
Akt	Akt serine/threonine kinase 1
AMBRA1	Activating Molecule in Beclin-1-Regulated Autophagy 1
AMPK	AMP-activated protein kinase
ATG proteins	Autophagy related proteins
ATG13	Autophagy related 13
BRCA1/2	Breast cancer type 1/2 susceptibility protein
DAMPs	Damage-associated molecular patterns
DIRAS3	GTP-binding protein Di-Ras3
DRAM1	DNA damage-regulated autophagy modulator 1
EGFR	epidermal growth factor receptor
EMT	epithelial–mesenchymal transition
ERK1/2	Extracellular Signal-Regulated Kinase 1 and 2
ER	endoplasmic reticulum
FIP200	FAK family kinase-interacting protein of 200 kDa
FRα	folate receptor alpha
GAPs	GTPase-activating proteins
GDP	guanosine diphosphate
GEFs	Guanine nucleotide exchange factors
GTP	guanosine triphosphate
HMGB1	High Mobility Group Box 1
HO-1	heme oxygenase 1
HPV	human papillomavirus
hTERT	human telomerase reverse transcriptase
KEAP1	kelch-like ECH-associated protein
K-RAS	Kirsten rat sarcoma viral oncogene homolog protein
LC3	microtubule-associated protein 1A/1B-light chain 3
MCL1	Myeloid Cell Leukemia-1
mTOR	mammalian target of rapamycin
mTORC1	mTOR kinase complex 1
NFKB1	Nuclear Factor Kappa B Subunit 1
NLRP3	NLR family pyrin domain containing 3
NOTCH1	Neurogenic locus notch homolog protein 1
NRF2	Nuclear factor erythroid 2-related factor 2
OC	ovarian cancer
OCSCs	ovarian cancer stem cells
p21	cyclin-dependent kinase inhibitor 1
p27	cyclin-dependent kinase inhibitor p27
p53	Tumor Protein 53
p62	ubiquitin-binding protein p62
PARP	poly(ADP-ribose) polymerase
PI3K	phosphoinositide 3-kinase
PIP2	phosphatidylinositol-4,5-bisphosphate
PIP3	phosphatidylinositol-3,4,5-triphosphate
pRb	retinoblastoma protein
PTEN	Phosphatase and tensin homolog
RAB	Ras-associated binding proteins
RAD51	DNA repair protein RAD51 homolog 1
RAP80	Receptor-associated protein 80
RNF168	Ring finger protein 168
ROS	reactive oxygen species
shRNA	short hairpin RNA
SNARE	soluble NSF attachment protein receptors
TFEB	transcription factor EB
TXNDC17	Thioredoxin domain-containing protein 17
UBE2T	ubiquitin-conjugating enzyme E2T
ULK1	Unc-51 like autophagy activating kinase 1
VEGF	vascular endothelial growth factor
VPS34	phosphatidylinositol 3-kinase VPS34
Wt–p53	wild type Tumor Protein 53
